# Understanding stress resilience

**DOI:** 10.3389/fnbeh.2013.00158

**Published:** 2013-11-08

**Authors:** Michael V. Baratta, Robert R. Rozeske, Steven F. Maier

**Affiliations:** ^1^Institute for Behavioral Genetics, University of Colorado BoulderBoulder, CO, USA; ^2^Neurocentre Magendie, INSERM U862Bordeaux, France; ^3^Department of Psychology and Neuroscience, University of Colorado BoulderBoulder, CO, USA

**Keywords:** resilience, stress, trauma, coping, depression, anxiety, posttraumatic stress disorder, neuroimaging

Adverse events can impact brain structure and function and are considered primary sources of risk for depression, anxiety, and other psychiatric disorders. However, the majority of individuals who encounter adverse or stressful life events do not develop untoward outcomes, and so an understanding of the factors that promote resistance to the deleterious effects of stress is of clinical importance. At the level of basic research, there has been considerable effort directed at identifying experimental parameters that blunt/augment outcomes from an adverse event, but even when parameters are held constant, there is inter-subject heterogeneity in behavior. This has shifted the focus to understanding how genetic and experiential factors can shape an organism's resistance to future adversity. The articles collected in the present Research Topic provide an overview of recent efforts directed at elucidating the neural mechanisms underlying resilience, and utilizing such information to mitigate vulnerability.

Achieving genetic, epigenetic, and neural circuit-level insight into the causal mechanisms underlying stress resilience has come from a variety of disciplinary approaches. Wu et al. (2013) lead this special issue by providing a comprehensive overview of recent progress in each of these units of analysis. At the human level, much of what is known about the biological determinants of resilience has increasingly come from neuroimaging studies. In this issue van der Werff et al. (2013) examine the structural and functional alterations related to resilience, particularly contrasting findings of individuals who either have, or have not, developed posttraumatic stress disorder (PTSD) following trauma.

One of the consequences of long-term stress exposure is to disrupt processes involved in successful adaptation to environmental threat. The endogenous opioid peptide, β-endorphin, has been shown to facilitate recovery following stress and here Barfield et al. (2013) directly investigate its role in anxiety-like behaviors using transgenic mice with varying capacities to synthesize the peptide. Vander Weele et al. (2013) examine the contribution of another endogenous peptide, growth hormone (GH), in hippocampal dysfunction following prolonged stress. The authors demonstrate that chronic stress regimens reduce hippocampal GH and that stress-induced hippocampal-dependent learning impairments are restored following site-specific viral-mediated overexpression of GH. Their findings implicate GH signaling in the hippocampus as a novel target for promoting resilience following prolonged stress regimens.

Environmental conditions during development also contribute to the heterogeneity of an individual's response to adversity encountered as an adult. As Macrì (2013) outlines, in an opinion piece, developmental conditions may be harnessed to promote resilience in experimental settings. One such experiential factor in humans, access to regular physical activity, has long been known to positively modulate an individual's adaptive capacity in the face of stress, an outcome that is readily observed with voluntary wheel running in rodents (Greenwood and Fleshner, 2011). Here Loughridge et al. (2013) combine laser capture microdissection with microarray expression analysis to investigate novel molecular targets of exercise-induced stress resistance. In addition to several genes that participate in neural mechanisms previously shown to be critical in the impact of exercise on stress, their innovative approach revealed sets of immune- and circadian-related genes that deserve further investigation.

In many organisms, prior exposure to repeated stressors often potentiates the neural and behavioral responses to later adverse events, a phenomenon termed stress sensitization, which is thought to be an important process involved in the susceptibility to anxiety disorders. Conversely, a reduction in autonomic, neuroendocrine, and behavioral responses is observed following repeated exposures to identical stressors (homotypic stress), a phenomenon termed stress habituation. Herman (2013) discusses the mechanisms underlying habituation and the challenges of identifying neural processes that represent a transition from adaptive to maladaptive responding during conditions of chronic challenge.

Psychological and social factors (e.g., coping style, cognitive flexibility, and social support) have long been associated with resilience to adversity (Southwick et al., 2005), although many of these factors are difficult to study in animals where the underlying neural mechanisms can be directly explored. In an animal model of intimate partner violence, Poirier et al. (2013) examine the influence of baseline trait anxiety in female rats on the neural and behavioral consequences of long-term cohabitation with an aggressive male partner. Despite the fact that anxious temperament modulated the behavioral outcome, many of the regional gene expression patterns that are altered by cohabitation did not differ between the “low” and “high” anxiety subgroups, highlighting the growing consensus that mechanisms of resilience are not always the opposite of those mediating vulnerability.

Additional psychosocial factors associated with resilience involve processes that engage coping strategies (Agaibi and Wilson, 2005). Active coping is generally conceptualized as behavioral or psychological efforts that individuals employ to master or reduce negative circumstances. Actual or perceived behavioral control over some aspect of the adverse event is central to coping, and Drugan et al. (2013) provide a review of rodent paradigms in which the degree of behavioral control is experimentally manipulated. The authors further discuss the development of continuous non-invasive measurements (e.g., ultrasonic vocalizations) within the original stress episode that may be predictive of later performance during a subsequent challenge. On a related note, Nechvatal and Lyons (2013) review how coping, within the context of stress exposure therapy, impacts functional and structural measures in patients with specific phobias or PTSD. A long-term goal of this line of research, the authors note, is to guide the development of new intervention modalities that enhance the neuroadaptations associated with coping with stress in order to facilitate recovery.

Overall, the perspectives presented in this *Frontiers in Behavioral Neuroscience* Research Topic represent an integrative approach for elucidating the neural mechanisms underlying stress resilience. Extension of these efforts to other experimental paradigms may identify common themes that will very likely inform and enhance current therapeutic modalities.
